# Focus on the Lung

**Published:** 2010

**Authors:** David Quintero, David M. Guidot

**Keywords:** Alcohol abuse, chronic alcohol effect, human immunodeficiency virus-1, lung, lung function, pneumonia, pulmonary diseases

## Abstract

Both HIV-1 infection and chronic alcohol abuse adversely affect lung health. For example, through multiple mechanisms, chronic alcohol abuse increases one’s susceptibility to pneumonia, particularly pneumonia caused by certain serious pathogens. Similarly, pneumonia caused by opportunistic pathogens is very common in HIV-infected patients, at least in part because HIV-1 attacks the immune cells of the lungs and interferes with their functions. Alcohol abuse also increases the risk of developing acute respiratory distress syndrome, a serious acute lung condition; however, the association of this syndrome with HIV-1 infection remains unclear. Chronic lung conditions potentially caused or exacerbated by chronic alcohol abuse include asthma, emphysema, or chronic bronchitis, although the findings to date are equivocal. However, growing evidence indicates that HIV-1 infection increases the risk of chronic pulmonary diseases such as emphysema, lung cancer, and excessive blood pressure in the vessels supplying the lung (i.e., pulmonary hypertension). Both alcohol abuse and HIV infection can impair lung function through various mechanisms, including increasing oxidative stress and enhancing antioxidant deficits, preventing full activation of the lung’s immune cells, and contributing to zinc deficiency. However, the interactions between alcohol abuse and HIV-1 infection in contributing to the range of lung disorders have not been studied in detail.

Despite considerable experimental and clinical evidence that both human immunodeficiency virus (HIV)-1 infection and chronic alcohol abuse render people susceptible to serious lung infections (i.e., pneumonia), surprisingly little information is available regarding the interactions between these two conditions on lung health. The same is true for lung diseases such as asthma, emphysema,^1^ chronic bronchitis, lung cancer, and pulmonary hypertension, which are not caused by infections but result from other disease processes. Thus, in most cases, little or no specific evidence suggests that the effects of concurrent HIV-1 infection and alcohol abuse are as great as or even greater than the sum of the effects of both individual conditions (i.e., are additive or synergistic) on these common noninfectious lung diseases in humans. However, findings from experimental animal models provide compelling reasons to infer that alcohol abuse has serious consequences on lung health in HIV-1–infected people. Moreover, recent discoveries in experimental models that complement researchers’ understanding of the pathophysiology of HIV-1 infection and alcohol abuse in humans have provided clues to potential new therapies for patients with infectious and noninfectious lung disease. This article summarizes some of the experimental and clinical evidence that implicate alcohol abuse as a potential exacerbating factor on lung health in HIV-1–infected people.

## Alcohol, HIV-1 Infection, and Pneumonia

Alcohol is the most frequently abused drug in the world (Lieber 1995). In the United States, 50 percent of the population regularly consumes alcohol, and in 2002 nearly 18 million American adults met the clinical diagnostic criteria for alcohol abuse or alcohol dependence (Grant et al. 2004). According to the most recent definition established by the American Psychiatric Association (2000) in the *Diagnostic and Statistical Manual of Mental Disorders, 4th Edition, Text Revision* (DSM–IV–TR), substance use disorders, which have been separated into substance abuse and substance dependence, are characterized by repeated use despite adverse social and/or physical consequences. Dependence has been considered a more advanced stage of a substance use disorder with specific tolerance and withdrawal phenomena. In other words, abuse is characterized by the use of a substance with psychoactive properties either in socially inappropriate ways or in spite of serious medical, legal, or social consequences, such as disruption of one’s personal or professional life. In contrast, dependence implies a state in which, in addition to the signs and symptoms of abuse, sudden withdrawal of the substance produces significant biological consequences. Thus, considerable overlap exists between alcohol abuse and alcohol dependence, and it is important to realize in this context that a drinker may suffer significant biological consequences of alcohol abuse (e.g., alcohol-related lung disease) even if he or she does not exhibit features of dependence, such as delirium tremens or other manifestations of a withdrawal syndrome. Further, it is important to note that alcohol abuse is not defined by the quantity of alcohol consumed but rather by the harmful consequences of its consumption. As a result, there is no consensus in clinical studies or experimental animal models on how to define alcohol abuse or even (harmful) chronic alcohol ingestion. Although this lack of standardization can complicate the interpretation of such studies, broad consensus exists among researchers and clinicians that chronic alcohol ingestion in excess of some safe threshold (which may vary among individuals) clearly is linked to multiple health problems. Unless otherwise specified, in this article, the term “alcohol abuse” refers to any type of alcohol use that results in adverse medical, legal, or social effects, regardless of whether it also meets the criteria for alcohol dependence.

HIV-1–infected populations in the United States, and throughout the world, show disproportionately high rates of alcohol abuse compared with the general population, which complicates all aspects of medical care for these patients (Fisher et al. 2007; Fortenberry 1998; Martin 1990). Moreover, experimental evidence in nonhuman primate models of the closely related simian immunodeficiency virus (SIV) suggests that chronic alcohol ingestion accelerates the natural history of the viral infection and the emergence of certain uncommon infections (i.e., opportunistic infections) (Bagby et al. 2003). This is not surprising because even in the absence of HIV-1 infection, alcohol abuse is an important risk factor for bacterial pneumonias (Fernandez-Sola et al. 1995). (For recent reviews of this topic, see Gamble et al. 2006; Happel and Nelson 2005; Zhang et al. 2002.) In fact, alcohol abuse has been recognized as a major risk factor for pneumonia for centuries, as evidenced by opinions from Benjamin Rush published in 1785 and Sir William Osler in 1905 (reviewed in Joshi and Guidot 2007; Nelson and Kolls 2002). Alcohol abuse reduces the ability of the lungs to defend themselves against infection, thereby rendering people susceptible to pneumonia through various mechanisms, including the following (for a review, see Joshi and Guidot 2007):

- Changes in the normal bacterial populations in the mouth and throat (i.e., the oropharyngeal flora);
- Decreased mucociliary function—that is, reduced activity of the fine hairs (i.e., cilia) that, through their movement, help remove mucus and inhaled particles from the conducting airways; and
- Impaired innate and adaptive immune responses to pathogens within the lower airways.

Detailed analyses of the causative bacteria also found that alcoholics are more likely to become infected with certain serious pathogens, such as *Klebsiella pneumoniae* (Jong et al. 1995), and to develop severe consequences (e.g., bacteremia and shock) even from typical pathogens, most notably *Streptococcus pneumoniae* (de Roux et al. 2006; Perlino and Rimland 1985), which in nonalcoholic patients typically cause less severe manifestations of the disease. However, to our knowledge, there is no evidence that alcohol abuse increases the risk of pneumonia caused by opportunistic pathogens that characteristically are associated with HIV-1 infection, such as *Cryptococcus neoformans* or *Pneumocystis jirovecii*.

Research also has shown that lung infections from opportunistic as well as typical pathogens remain a major cause of morbidity and mortality in HIV-1–infected people. Despite significant advances in HIV-1 treatment, including the development of highly active antiretroviral therapy (HAART), infected people still are prone to pneumonias caused by pathogens such as *Klebsiella pneumoniae* and *Mycobacterium tuberculosis* (Afessa and Green 2000*b*; Shankar et al. 2006). In addition, HIV-1–infected people are at increased risk for more community-acquired pneumonias caused by more typical pathogens, including *Streptococcus pneumoniae*^2^ and *Haemophilus influenza* (Afessa and Green 2000*a*; Lin and Nichol 2001), suggesting that the viral infection weakens the lung’s defenses against subsequent bacterial infections.

The precise mechanisms underlying HIV-1–induced immune dysfunction within the lung are not known. Within the lower airways, the primary innate immune defenses depend on a type of immune cells known as alveolar macrophages, which recognize bacteria, viruses, and other foreign particles; engulf them; and then destroy them in a process called phagocytosis. To exert these functions, the alveolar macrophages interact with molecules (i.e., factors) secreted by other cells, including so-called surfactant proteins. Although the precise mechanisms still are being investigated, it has been shown that HIV-1 can infect alveolar macrophages (Stebbing et al. 2004); moreover, considerable evidence indicates that phagocytosis and other innate immune functions are impaired in macrophages and their precursor cells (i.e., monocytes) from HIV-1–infected patients (Kedzierska et al. 2003; Noursadeghi et al. 2006; Pugliese et al. 2005; Thomas and Limper 2007). Finally, in an experimental rodent model, HIV-1 infection decreases phagocytosis and increases the severity of lung infection caused by the pathogen *Mycobacterium tuberculosis* (Mason et al. 2004).

Together, these observations indicate that both HIV-1 infection and alcohol abuse render people susceptible to lung infections, increasing the incidence and severity of common infections such as tuberculosis and pneumococcal pneumonia. To our knowledge, however, no epidemiological studies have been performed to determine whether alcohol abuse increases the risk and/or severity of lung infections in HIV-1–infected patients. Such studies would be extremely difficult to perform and would be complicated by issues such as underlying smoking, nonadherence to antiretroviral therapy, and malnutrition. One opportunity to determine such associations may be through analyses of existing databases, such as the Veterans Aging Cohort Study, using retrospect to try to identify an independent effect of alcohol abuse on specific outcomes such as pneumonia in HIV-1–infected people. Interestingly, some evidence obtained in rodent models suggest that alcohol ingestion decreases lung host defense against an opportunistic pathogen called *Pneumocystis carinii*, which commonly is found in HIV-infected patients (D’Souza et al. 1995), and this issue merits further investigation.

## Alcohol, HIV-1 Infection, and Noninfectious Pulmonary Diseases

In addition to being associated with an increased risk of lung infections caused by a variety of pathogens, alcohol abuse and HIV-1 infection each appear to alter the risk of both acute and chronic noninfectious lung diseases, as discussed in the following paragraphs.

### Acute Lung Disease

#### Impact of Alcohol Abuse

In the past decade, researchers and clinicians have recognized that alcohol abuse significantly increases the risk of a sudden, serious, or even life-threatening condition called acute respiratory distress syndrome (ARDS). In patients with this condition, the small air-filled sacs (i.e., alveoli) at the end of the airways in the lungs become inflamed, fill with fluid, and collapse (for an illustration of the anatomy of the airways and lungs, see the figure). As a result of this fluid accumulation (i.e., edema) and alveolar collapse, the exchange of oxygen and carbon dioxide that is the central function of the lung can no longer occur and the body becomes starved for oxygen. ARDS develops in response to a variety of conditions that result in inflammatory responses, including sepsis, trauma, inhalation of stomach contents into the airways (i.e., gastric aspiration), pneumonia, and massive blood transfusions (Ware and Matthay 2000).

The alveolar walls are made up of two types of cells that both are affected by ARDS: (1) cells that form a tight barrier preventing any fluid from entering the alveoli and through which the actual gas exchange occurs, and (2) cells that secrete surfactant protein, which is essential for preventing the alveoli from collapsing. Originally described by Ashbaugh and colleagues (1967), ARDS is characterized by disruption of the cell barrier and surfactant dysfunction as well as by an intense inflammation. These factors in concert produce profound derangements in gas exchange and severe respiratory failure. Although much has been learned about the underlying pathophysiology of ARDS in the past four decades, treatment remains essentially supportive, and even with aggressive care the mortality rate of patients with this syndrome remains unacceptably high at 40 to 60 percent (Rubenfeld et al. 2005; Ware and Matthay 2000).

Recent epidemiological studies have provided an important clue as to which patients are at increased risk for ARDS by demonstrating a link between alcohol abuse and acute lung injury. A landmark study by Moss and colleagues (1996) first identified an independent connection between alcohol abuse^3^ and ARDS, with 51 percent of the ARDS patients classified as alcoholics. A subsequent prospective study (Moss et al. 2003) in 220 patients with septic shock confirmed this initial observation, with 49 percent of the patients who developed ARDS being classified as alcoholics.^4^ The latter study also concluded that the risk of ARDS was 3.7 times higher in alcoholic patients than in nonalcoholic patients when all other factors were taken into consideration. If these findings are extrapolated to the population at large, then alcohol abuse is associated with the development of ARDS in tens of thousands of patients in the United States each year. This extrapolation is supported by a decade of experimental evidence in laboratory models showing that chronic alcohol ingestion renders the lung susceptible to injury. For example, in rodent models, alcohol ingestion for just 6 weeks decreases levels of the antioxidant glutathione in the airways and increases the severity of lung injury in response to a bacterial toxin (i.e., endotoxin) or bacterial sepsis (Holguin et al. 1998; Velasquez et al. 2002). For more comprehensive reviews of the clinical and experimental evidence linking alcohol and lung injury, the reader is referred to reviews by Guidot and Hart (2005) and Joshi and Guidot (2007).

#### Impact of HIV-1 Infection

In contrast, a link between HIV-1 infection and an increased risk for acute lung injury has not been established because no comparable epidemiological studies have been conducted. In fact, epidemiological and clinical intervention studies in acute lung injury have consistently excluded people with HIV-1 infection from enrollment. Therefore, although severe pneumonia and its associated respiratory failure remains the leading cause of death in HIV-1–infected people, it is unknown if HIV-1 infection independently increases the risk of acute lung injury. New experimental evidence suggests, however, that HIV-1–related proteins cause oxidative stress^5^ and induce dysfunction of the alveolar cell barrier (these effects are discussed further below). Thus, it would seem that an acute insult (e.g., trauma or septic shock) would be much more likely to lead to ARDS in people with underlying HIV-1 infection than in people without such an infection. Future epidemiological studies that include HIV-1–infected participants may resolve this question.

Other findings suggest that in addition to potentially increasing the risk of acute lung injury, underlying HIV-1 infection also may be associated with a worse outcome in patients with acute lung injury. Thus, at least two studies (Suchyta et al. 1992; Torres et al. 1995) have demonstrated that HIV-1 infection independently increases mortality rates in patients with acute lung injury.

### Chronic Lung Disease

#### Impact of Alcohol Abuse

In contrast to the well-established link between alcohol abuse and acute lung injury, the existence of an independent connection between lung function or chronic airway disease and alcohol consumption has been controversial:

- In an epidemiological study of 1,067 men evaluated over a 5-year period, alcohol ingestion did not significantly affect baseline or 5-year follow-up pulmonary function tests after controlling for height, smoking, and education level (Sparrow et al. 1983).
- In a larger 5-year study involving 11,135 participants, patients with heavy alcohol consumption had an accelerated loss of lung function over time and one measure of lung function (i.e., the forced expiratory volume in 1 second [FEV_1_]) was decreased as much as by smoking 15 cigarettes per day (Lange et al. 1988).
- Excessive alcohol intake has been associated with increased prevalence of airway obstruction in the general population, and Sisson and colleagues (2005) reported that former heavy drinkers are twice as likely as others to have airway obstruction after adjusting for cigarette smoking.
- Chronic ingestion of alcohol, particularly of distilled spirits, has been linked to certain chronic pulmonary conditions including emphysema, fibrosis, and bronchiectasis. It is difficult to determine whether these are associations or cause-and-effect relationships because a large proportion of alcohol abusers smoke cigarettes, which also contribute substantially to the development of chronic lung disorders. As early as 1967, however, Burch and DePasquale (1967) suspected that these diseases result, at least in part, from direct alcohol-induced damage of lung tissue and suggested the existence of an alcoholic lung disease.

To our knowledge, there have been no studies in animal models to determine whether chronic alcohol ingestion can independently cause emphysema, chronic bronchitis, and/or related airways disease.

Some observations suggest that chronic alcohol consumption may impact asthma, an airway disease not closely associated with cigarette smoking that is characterized by chronic airway inflammation and narrowing of the airways (i.e., bronchoconstriction), which leads to breathing difficulties. For example, alcohol consumption has been associated with increased bronchial hyperreactivity^6^ and increased frequency of severe asthma attacks (i.e., asthma exacerbations) (Cuddy and Li 2001; Kawano et al. 2004; Shimoda et al. 1996). On the other hand, in a review on alcohol and airway disease, Sisson (2007) concluded that alcohol is moderately effective in transiently widening the airways (i.e., is a transient bronchodilator) and likely relaxes airway smooth muscle tone following acute ingestion. However, in some people—including those who have a genetically determined decreased capacity to eliminate the alcohol breakdown product acetaldehyde (i.e., who have decreased activity of the enzyme aldehyde dehydrogenase)—the acetaldehyde that is generated after alcohol consumption and/or other components of alcoholic beverages may trigger bronchoconstriction and thus an asthma attack.

Another pathway through which chronic alcohol consumption may impact asthma patients is by altering the levels of oxidative stress and antioxidants. Asthma patients have increased baseline levels of oxidative stress in the airways, which normally are compensated by increased levels of the antioxidant glutathione (Fitzpatrick et al. 2009). Alcohol consumption is known to increase oxidative stress and lead to decreased glutathione levels. Therefore, in asthmatics who drink, alcohol ingestion may further increase baseline airway oxidative stress as well as blunt the normal compensatory increase in glutathione. Importantly, reductions in airway glutathione levels have resulted in increased bronchial hyperreactivity in experimental asthma models; therefore, alcohol’s effects on oxidative stress and antioxidants may be a critical pathway by which chronic alcohol consumption leads to increased asthma exacerbations (Koike et al. 2007). This is an important issue from a clinical and public health perspective. According to a nationally representative survey from the Centers for Disease Control and Prevention (CDC), the 2004 Behavioral Risk Factor Surveillance System (BRFSS), approximately 45 percent of the adult asthmatic population consume alcohol regularly. Among those who drink, approximately one-third (corresponding to 15 percent of the total asthmatic population, or more than 2.7 million people^7^) are considered at risk for heavy alcohol consumption (http://www.cdc.gov/brfss/).

#### Impact of HIV-1 Infection

Growing evidence indicates that HIV-1 infection increases the risk of chronic airway diseases such as emphysema. Among participants in the Veterans Aging Cohort Study, HIV-1–infected people were 50 to 60 percent more likely than others to develop chronic obstructive pulmonary disease (COPD), which includes emphysema and/or chronic bronchitis; moreover, HIV-1 infection was an independent risk factor for COPD even after accounting for age, race/ethnicity, smoking history, and substance abuse (Crothers et al. 2006). Other studies have corroborated these findings, and several recent reviews summarize the clinical data to date as well as some of the laboratory-based research that is beginning to explore possible mechanisms (Corthers 2007; Kanmogne 2005; Petrache et al. 2008).

One of the hallmarks of HIV-1 infection and acquired immune deficiency syndrome (AIDS) is the development of certain, otherwise rare, types of tumors (i.e., malignancies), such as Kaposi’s sarcoma. Since the introduction of HAART to treat chronic HIV infection, however, the rates of such AIDS-defining malignancies appear to have fallen dramatically, whereas the rates of other cancers, including lung cancer (i.e., bronchogenic carcinoma), actually are increased in the AIDS population (Bonnet and Chene 2008). In parallel, epidemiological studies have suggested that lifelong alcohol consumption may increase the risk of lung cancer beyond what would be expected from cigarette smoking alone. For example, a recent study from Montreal, Canada, found that people who consumed alcohol all their lives had an odds ratio of 1.6 for developing lung cancer (Benedetti et al. 2009). Therefore, although tobacco smoking clearly is the primary risk factor for lung cancer overall, there is evidence that both chronic alcohol ingestion and chronic HIV-1 infection may increase this risk.

Finally, HIV-1 infection clearly is associated with a dramatically increased risk of pulmonary hypertension, with an incidence that is several thousand times the rate among the general population (Seoane et al. 2001; Speich et al. 1991). Researchers have estimated that 0.5 to 1.0 percent of people with HIV-1 infection develop pulmonary hypertension, and the incidence appears to be increasing (Nunes et al. 2003). Scientists generally believe that several insults (or “hits”) to the pulmonary system must occur before a person develops pulmonary hypertension. Thus, it is possible that the increased lifespan of HIV-1–infected patients that results from more effective therapy increases the likelihood that patients are exposed to the multiple hits required to develop pulmonary hypertension (Yuan and Rubin 2005). In contrast to HIV-1 infection, alcohol abuse appears to be linked to pulmonary hypertension only indirectly, because some patients with alcohol abuse–related liver damage (i.e., cirrhosis) and elevated blood pressure in the vessels supplying the liver (i.e., portal hypertension) also develop severe pulmonary hypertension. Moreover, older clinical studies (Conway 1968; Gould et al. 1972) have suggested that acute alcohol consumption does not increase blood pressure in the vessels to and from the lung.

## How Do Alcohol Abuse and HIV-1 Infection Impair Lung Health?

As discussed earlier, the association between alcohol abuse and pneumonia has been recognized for centuries, and multiple mechanisms have been identified that underlie this association. For example, chronic alcohol abuse impairs the secretion of saliva, promotes gingivitis, and increases colonization of the mouth and throat (i.e., pharynx) with certain bacteria frequently associated with respiratory tract infections (Fuxench-Lopez and Ramirez-Ronda 1978; Happel and Nelson 2005). Because of these consequences of chronic alcohol consumption, as well as the well-known effects of acute intoxication (i.e., impaired consciousness and decreased gag reflex), alcoholics are more likely to suffer infections from pathogens into the lower airways. Moreover, alcohol appears to impair the body’s defense mechanisms when bacteria reach the windpipe (i.e., trachea) and conducting airways. Normally, the first defense mechanism involves removal of the pathogens by the mucus produced in the airways, which is swept out of the respiratory tract by the cilia lining the airways. Experimental animal models, however, have demonstrated that alcohol impairs this function (Wyatt et al. 2004). If pathogens reach the lower airways, including the alveoli, the next defense mechanism involves innate immune defenses that depend on the phagocytic functions of alveolar macrophages and their interaction with secreted factors, including surfactant proteins. As discussed earlier, experimental models have provided evidence that alcohol ingestion impairs the innate immune functions of alveolar macrophages. Consistent with these experimental findings, alveolar macrophages isolated from alcoholic patients exhibited impaired immune functions, including decreased secretion of a signaling molecule called tumor necrosis factor-α, which regulates the activities of various immune cells (Omidvari et al. 1998). Moreover, alcohol can decrease the ability of surfactants to facilitate macrophage phagocytosis and killing of *Streptococcus pneumoniae* bacteria in laboratory experiments (Rubins et al. 1996), which would exacerbate impairment of the innate immune response to infections in the alveolar space. Finally, the coordinated adaptive immune response to infection that must be initiated by the alveolar macrophages also is dampened because the macrophages produce fewer signaling molecules (i.e., chemokines) that normally recruit other immune cells (i.e., neutrophils) to the alveolar space to combat the bacterial infection (Boe et al. 2003; Quinton et al. 2005).

In this brief review, it is impossible to discuss all the potential biological mechanisms by which alcohol abuse and HIV-1 infection might interact to render people susceptible to pulmonary disease. However, three possibilities suggested by experimental models and clinical studies will be discussed here in more detail:

- Oxidative stress and glutathione depletion;
- Impaired “priming” of alveolar macrophages; and
- Zinc deficiency.

### Oxidative Stress and Glutathione Depletion

As mentioned earlier, oxidative stress refers to an imbalance between the levels of highly reactive oxygen–containing molecules and antioxidants, such as glutathione. People infected with HIV-1 show evidence of oxidative stress, particularly decreased glutathione levels, throughout their body (i.e., systemic oxidative stress) (Buhl et al. 1989; Eck et al. 1989; Treitinger et al. 2000). Interestingly, not all consequences of HIV-1 can be attributed to direct viral infection; instead, virus-related proteins produced by the infected cells also may contribute to the pathophysiology of the disease. For example, compelling experimental data indicate that HIV-1–related proteins, particularly a protein called Tat,^8^ can induce significant oxidative stress even in cells that are not infected with the virus. Such effects can be studied in genetically modified laboratory animals (i.e., transgenic animals) that produce (i.e., express) specific HIV-1–related proteins but are not infected with the entire virus and do not show viral replication. Researchers recently reported that transgenic expression of HIV-1–related proteins in mice and rats caused severe oxidative stress in the lung, including glutathione depletion, and impaired the barrier function of the alveoli (Kline et al. 2008; Lassiter et al. 2009). These findings are remarkably similar to the effects of chronic alcohol ingestion on lung glutathione and alveolar epithelial barrier function that have been identified both in experimental models and in otherwise healthy people who abuse alcohol (Guidot et al. 2000; Holguin et al. 1998; Moss et al. 2000). Importantly, dietary supplementation with glutathione precursors mitigates the effects of both transgenic expression of HIV-1–related proteins and chronic alcohol ingestion in animal models (Guidot et al. 2000; Holguin et al. 1998; Kline et al. 2008; Velasquez et al. 2002), suggesting that such dietary supplements could be beneficial as complementary therapy in humans.

### Impaired “Priming” of Alveolar Macrophages

As mentioned earlier, the airways’ defense system against infection and other harmful influences involves both innate and adaptive immune responses, with alveolar macrophages playing a central role in the innate immune response. To fully exert their function, these macrophages first must be “primed” by being exposed to a body-produced factor called granulocyte/macrophage colony–stimulating factor (GM-CSF) that binds to docking molecules (i.e., receptors) on the macrophage’s surface. Studies found that both transgenic expression of HIV-1–related proteins and chronic alcohol ingestion impair alveolar macrophage priming by GM-CSF, including decreased membrane expression of the GM-CSF receptor (Joshi et al. 2005). As a result, GM-CSF signaling is significantly dampened. Additional studies in an experimental model of chronic alcohol ingestion demonstrated that treatment with externally produced (i.e., recombinant) GM-CSF can restore the functions of both the alveolar macrophages and the alveolar epithelium (Joshi et al. 2005, 2006; Pelaez et al. 2004), suggesting that GM-CSF administration might be effective in the acute setting when alcoholic patients are admitted to the hospital with serious illnesses such as pneumonia. However, whether GM-CSF treatment can improve alveolar macrophage or epithelial function in the context of HIV-1 infection has not yet been evaluated.

### Zinc Deficiency

A common link that may unify the observed oxidative stress and impaired immune function in the airways caused by alcohol abuse and HIV-1 infection could be zinc deficiency. Zinc is the most abundant trace metal in the body other than iron and is critically involved in cell growth, differentiation, and function. For example, zinc serves as a cofactor for about 300 known enzymes, including several key antioxidants, and also is vital for the function of thousands of factors that regulate gene activity (i.e., transcription factors). Zinc deficiency produces widespread abnormalities but is particularly detrimental to epithelial cells and to the immune system. Zinc deficiency contributes to disease burden in many areas, most notably sub-Saharan Africa and Southeast Asia (Tudor et al. 2005). In the United States, dietary zinc deficiency is less common but remains problematic in people with either a zinc-poor diet or other abnormalities that limit zinc absorption or sequester zinc in the liver as a response to an infection. Importantly, chronic alcohol abuse has a strong association with zinc deficiency. Although some of this zinc deficiency likely results from poor dietary intake, Joshi and colleagues (2009) recently reported that in rats, chronic alcohol ingestion^9^ causes a previously unrecognized inhibition of a molecule that transports zinc across the walls of the intestine and the lungs (i.e., the zinc importer ZIP4). Remarkably, when the animals received dietary zinc supplements at doses comparable with those used to treat severe zinc deficiency in humans, the deleterious effects of chronic alcohol ingestion on GM-CSF signaling and bacterial phagocytosis in the alveolar macrophage were reversed (Joshi et al. 2009).

Similarly, a higher incidence of bacterial infections has been reported in HIV-1–infected people with low zinc levels (Koch et al. 1996); moreover, lower levels of zinc correlate with more advanced stages of the disease (Allavena et al. 1995; Graham et al. 1991). Whether this deficiency is because of poor dietary intake or inhibition of zinc uptake into the body, like the one caused by alcohol ingestion, is unknown. However, similar to the effects observed in rats that chronically consume alcohol, dietary zinc supplementation restored GM-CSF signaling and alveolar macrophage phagocytic function in rats that were HIV-1 transgenic (Joshi et al. 2008). Taken together, these experimental findings suggest the intriguing possibility that dietary zinc supplementation could enhance pulmonary host defenses in the setting of alcohol abuse and/or HIV-1 infection. Clearly, this hypothesis can be tested in human patients, and clinical intervention studies with dietary zinc and/or other supplements (e.g., glutathione precursors) will no doubt be performed in the near future.

## Potential Clinical Implications of Alcohol Ingestion in People With HIV-1 Infection

As discussed in the preceding section, considerable circumstantial evidence suggests that chronic alcohol abuse (and perhaps even chronic moderate consumption) could exacerbate the oxidative stress and immune dysfunction in the airways of HIV-1–infected people. For example, the combined effects of chronic alcohol and HIV-1–related proteins on glutathione pools within the alveolar space may well increase the risks of acute lung injury and emphysema, both of which are characterized by damage to the alveolar epithelium. Similarly, zinc deficiency and the subsequent dampening of GM-CSF signaling within the alveolar space are features of both chronic alcohol ingestion and HIV-1–related protein expression in animal models. If these findings can be confirmed in future clinical studies, they will provide novel insights into the increased risk of serious pneumonia in HIV-1–infected, alcohol-consuming people as well as suggest potential new complementary treatments. These and other direct biological interactions between alcohol abuse and HIV-1 infection would further exacerbate the more indirect interactions, such as malnutrition and lack of adherence to HAART, and would likely result in impaired lung health in these patients. Further epidemiological studies are needed to clarify the precise relationship between alcohol abuse and HIV-1 infection on lung health. Perhaps more importantly, the experimental evidence for the common biological mechanisms by which alcohol abuse and HIV-1 infection impair host immunity and airway integrity in the lung can be used to design treatments that supplement standard antiretroviral therapy and improve lung health in these vulnerable people.

## Figures and Tables

**Figure:**
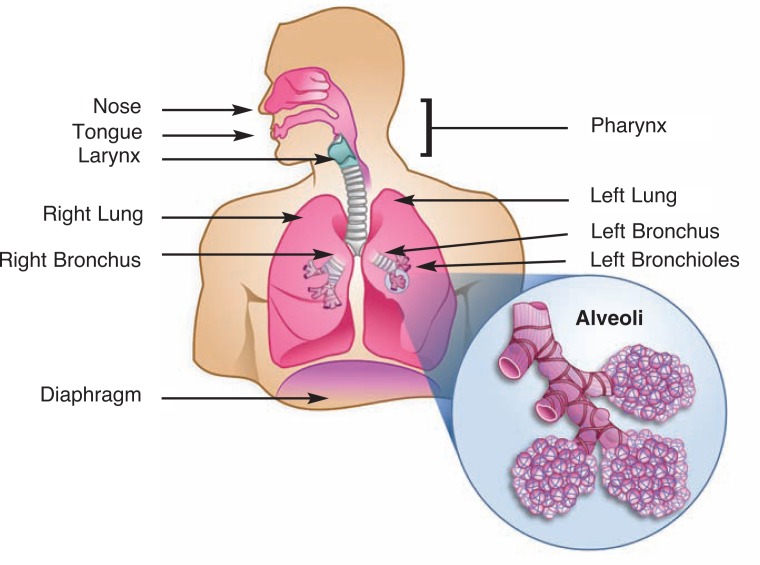
The human respiratory system.

## Footnotes

1 For a definition of this and other technical terms, see the Glossary, pp. 288–291.

2 Pneumonia caused by *Streptococcus pneumoniae* also is commonly referred to as pneumococcal pneumonia.

3 In the study by Moss and colleagues (1996), a diagnosis of chronic alcohol abuse was made if the patient had either been diagnosed with chronic alcoholism, had a history of admission to an alcohol detoxification center, or had previously been hospitalized for alcohol withdrawal.

4 In the study by Moss et al. 2003, a more standard definition of alcohol abuse using the Short Michigan Alcohol Screening Test was used to classify subjects as alcoholic versus nonalcoholic.

5 Oxidative stress is a state characterized by excessive levels of highly reactive and toxic oxygen-containing molecules and/or inadequate levels of agents (i.e., antioxidants) that can detoxify the reactive oxygen molecules, such as glutathione or vitamin E.

6 Bronchial hyperreactivity is a state characterized by easily triggered contractions (i.e., spasms) of the smallest branches of the airways, the bronchioles. Bronchial hyperreactivity is a hallmark of asthma but also often occurs in people with other diseases of the airways, such as chronic obstructive pulmonary disease.

7 This number is based on estimates of the New York State Health department that there are more than 18 million adult asthmatics in the United States (http://www.health.state.ny.us/statistics/ny_asthma/asthmaprevalence06.htm).

8 Tat is a regulatory protein encoded by the HIV-1 virus that accelerates the production of more virus particles by the infected cell; in fact, HIV-1 variants that encode no active Tat protein cannot replicate. In addition, the Tat protein is a toxin, causing the death of uninfected cells that take it up from the blood stream.

9 The animals were kept for 6 weeks on a liquid diet in which alcohol contributed 36 percent of the total calories but that otherwise was nutritionally complete.
